# Unveiling Molecular Signatures in Light-Induced Seed Germination: Insights from PIN3, PIN7, and AUX1 in *Arabidopsis thaliana*

**DOI:** 10.3390/plants13030408

**Published:** 2024-01-30

**Authors:** Rocío Soledad Tognacca, Karin Ljung, Javier Francisco Botto

**Affiliations:** 1 Instituto de Investigaciones Fisiológicas y Ecológicas Vinculadas a la Agricultura (IFEVA), Consejo Nacional de Investigaciones Científicas y Técnicas (CONICET), Facultad de Agronomía, Universidad de Buenos Aires (UBA), Buenos Aires C1417DSE, Argentina; 2 Departamento de Fisiología, Biología, Molecular, y Celular, Facultad de Ciencias Exactas y Naturales, Universidad de Buenos Aires, Buenos Aires C1428EGA, Argentina; 3 Instituto de Fisiología, Biología Molecular y Neurociencias (IFIBYNE), Consejo Nacional de Investigaciones Científicas y Técnicas (CONICET), Universidad de Buenos Aires, Buenos Aires C1428EHA, Argentina; 4 Umeå Plant Science Centre, Department of Forest Genetics and Plant Physiology, Swedish University of Agricultural Sciences, SE-901 83 Umeå, Sweden; karin.ljung@slu.se

**Keywords:** seed germination, auxin, ABA, hormonal crosstalk, PIN3, PIN7, AUX1, molecular regulation, *Arabidopsis thaliana*

## Abstract

Light provides seeds with information that is essential for the adjustment of their germination to the conditions that are most favorable for the successful establishment of the future seedling. The promotion of germination depends mainly on environmental factors, like temperature and light, as well as internal factors associated with the hormonal balance between gibberellins (GA) and abscisic acid (ABA), although other hormones such as auxins may act secondarily. While transcriptomic studies of light-germinating *Arabidopsis thaliana* seeds suggest that auxins and auxin transporters are necessary, there are still no functional studies connecting the activity of the auxin transporters in light-induced seed germination. In this study, we investigated the roles of two auxin efflux carrier (PIN3 and PIN7) proteins and one auxin influx (AUX1) carrier protein during *Arabidopsis thaliana* seed germination. By using next-generation sequencing (RNAseq), gene expression analyses, hormonal sensitivity assays, and the quantification of indole-3-acetic acid (IAA) levels, we assessed the functional roles of PIN3, PIN7, and AUX1 during light-induced seed germination. We showed that auxin levels are increased 24 h after a red-pulse (Rp). Additionally, we evaluated the germination responses of *pin*3, *pin*7, and *aux*1 mutant seeds and showed that PIN3, PIN7, and AUX1 auxin carriers are important players in the regulation of seed germination. By using gene expression analysis in water, fluridone (F), and ABA+F treated seeds, we confirmed that Rp-induced seed germination is associated with auxin transport, and ABA controls the function of PIN3, PIN7, and AUX1 during this process. Overall, our results highlight the relevant and positive role of auxin transporters in germinating the seeds of *Arabidopsis thaliana*.

## 1. Introduction

Seed dormancy plays a crucial role in enabling plant populations to adapt to their environment. The timing of seed germination is intricately shaped by environmental cues that influence the depth of dormancy and its alleviation, highlighting their paramount adaptive significance [1]. Key environmental factors, including temperature, light, and nitrates, govern the relief of seed dormancy, ultimately facilitating the germination of the seed population [2]. 

Light plays a pivotal role in the relief of dormancy and in the timing of seed germination. Phytochromes, which are well-established photoreceptors responsive to Red (R) and Far-Red (FR) light are synthesized in their inactive Pr form, which effectively absorbs R photons to photo-convert them into the active Pfr form, with maximum absorption in FR, which is the form that induces seed germination [3]. The *Arabidopsis thaliana* genome encodes five phytochromes, known as phyA to phyE, all of which have different functions in the control of seed germination [4,5]. The phyA- and phyB-dependent inductions of germination are spatially separated in the endosperm and embryo [6]. The molecular mechanisms of light which induce germination are well established [7]. In the dark, PHYTOCHROME INTERACTING FACTOR 1 (PIF1), also known as PHYTOCHROME INTERACTING FACTOR 3-LIKE 5 (PIL5), activates the expression of DELLAs, which represses gibberellic acid (GA) signaling by inhibiting GA biosynthetic and abscisic acid (ABA) catabolic genes; it also activates GA catabolic and ABA biosynthetic genes, which results in decreased GA levels and increased ABA levels. Upon R light perception, the Pfr form of the phytochromes binds to PIL5 and activates its degradation. The decrease in PIL5 reduces DELLAs and results in increasing GA and decreasing ABA levels [8,9,10]. Thus, both decreased ABA and increased GA levels are required for the relief of seed dormancy and subsequent germination. The finely tuned balance between hormone content and sensitivity in seed tissues (i.e., embryo and endosperm) governs the growth of the embryo during the germination process. Environmental signals contribute to the regulation of this balance by altering the expression of different metabolic enzymes, as well as the expression of positive and negative regulators of GA and ABA, many of which are subjected to feedback regulation [11].

Even though ABA and GA are the main hormones regulating dormancy and germination, other hormones, such as auxin, are also involved [12,13,14], though their roles are less understood [12]. Auxin can enter the cell through specialized transporters belonging to the AUXIN RESISTANT 1/LIKE AUX1 family (AUX1/LAX) [15]. The efflux of auxin relies on transporters from the PIN-formed (PIN) family or P-glycoprotein/ATP-binding cassette B (ABCB) transporters [16,17]. Notably, the most distinctive characteristic of the PINs is their polar distribution in the plasma membrane, enabling the directional flow of auxin. The action of auxin in seed dormancy requires the ABA signaling pathway (and vice-versa), suggesting that their roles in seed dormancy are interdependent [18]. Auxin enhances ABA signaling through *AUXIN RESPONSE FACTOR*10 *(ARF*10*)* and *ARF*16, functioning upstream of *ABI*3 [18,19], which, in turn, acts upstream of *ABI5* to execute ABA-responsive inhibition of seed germination [20]. The combined application of indole-3-acetic acid (IAA) and ABA dramatically inhibits seed germination, suggesting that ABA may exert its role through the auxin signaling pathway [18,21]. Additionally, *axr*2 and *axr*3 mutant seeds exhibit lower sensitivity to ABA compared to the wild-type, supporting the notion that ABA’s inhibitory effect on seed germination may occur through the auxin signaling pathway [18]. Conversely, the activation of auxin signaling leads to the binding of auxins to TIR1/AFB receptors, resulting in the degradation of AXR2 and AXR3, promoting the activity of ARF10 and ARF16, thus maintaining *ABI*3 expression [18]. Furthermore, ABA represses embryonic axis elongation by enhancing auxin signaling in the elongation zone of the radicle. The ABA-dependent repression requires auxin transport activity in the peripheral elongation zone and root cap through AUX1 and PIN2. ABA enhances auxin responses by downregulating *AXR2* expression in an auxin-independent manner [22].

Transcriptional studies demonstrate that phytochromes regulate hormone metabolism and signaling mainly through PIL5 during seed germination [9]. In imbibed seeds, PIL5 directly regulates auxin-related transcriptional regulators (*NIT1*, *IAA*16, and *ARF*18). For example, PIL5 activates the expression of *ARF*18, which encodes a positive signaling transcription factor, and represses the expression of the *IAA*16 gene, which encodes a negative signaling component [10]. Moreover, phyA positively regulates the expression of *ARF*18, *AXR*1, *AXR*4, *ATMYB*34, and auxin transporters like *PIN*1, *PIN*2, and *PIN*7 in *Arabidopsis thaliana* seeds [4]. However, some auxin metabolic genes, such as *CYP7*9*B*2 and *NIT*3, are repressed in the phyA-dependent germination [4]. These results are in accordance with another study that analyzed genes regulated by the PIL5-HFR1 module that mediates the phyB-dependent germination [23]. These authors showed that genes involved in auxin signaling and transport, such as *AUX*1/3, *PIN*1/2/3/7, *SUR*1/2, and *IAA*16, are promoted in light-treated seeds [23]. Despite the evidence, functional studies connecting the auxin transporter activity with the photocontrol of germination are still lacking.

In this study, we evaluated the relevance of the auxin transport during seed germination induced by a red-pulse (Rp) and mediated by the phyB. We showed that the auxin levels are increased 24 h after the Rp. Additionally, we demonstrated the functional relevance of the auxin efflux (PIN3 and PIN7) and influx (AUX1) carriers mediating Rp-induced seed germination. Furthermore, we showed that ABA inhibits their function, at least in part, by repressing *PIN*7, but not *PIN*3, and *AUX*1 gene expression, suggesting that ABA controls the function of the auxin transporters during light-mediated seed germination. Overall, our work highlights the relevance of the opposite function of auxin carriers in the crosstalk with the ABA signaling pathway during induction of dormancy and its positive role during seed germination. 

## 2. Results

### 2.1. Transcriptome Analysis Shows That Auxin Signaling Is Altered in R-Induced Seed Germination

**Figure 1:**
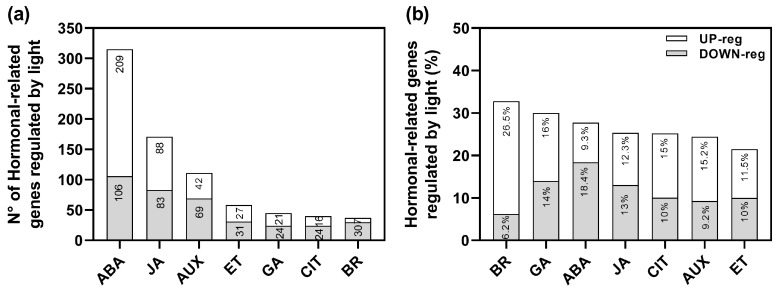
R-light regulates the expression of auxin-related genes in Col-0 seeds. (**a**) Number of genes related to hormonal pathways affected by light. (**b**) Percentage of hormone-related genes compared to Gene Annotation. The hormone-related gene annotation list was performed using the AmiGO 2 database. The % was calculated as (# of genes affected in our RNAseq as up or down-regulated/# of total genes annotated in each hormonal signature) × 100. See Supplementary Table S1 for more information. BR: brassinosteroids; GA: gibberellic acid; ABA: abscisic acid; JA: jasmonic acid; CIT: cytokinins; AUX: auxin; ET: ethylene (Data reanalyzed from [24]).

We have previously performed an RNA-sequencing (RNA-seq) analysis of imbibed wild-type Col-0 seeds in response to Red (Rp) and FR pulses (FRp) mediating the promotion and inhibition of germination [24]. We identified a total of 5785 genes significantly affected by light: 3099 genes were up-regulated whilst 2686 genes were down-regulated [24]. Based on this experiment, we reanalyzed the data to better understand the role of auxin-related genes that might be involved in the regulation of seed germination (Figure 1). The group of genes regulated by R-light was significantly enriched by hormonal genes (RF: 1.3; *p* < 4.22 × 10^−10^), representing 13.25% of the total transcriptome. The auxin signaling pathway was the third hormonal pathway significantly altered in response to R-light (111 genes; Figure 1a, Supplementary Table S1). These data confirm that auxins are important players in regulating R-induced seed germination.

The Gene Ontology enrichment analyses of the 111 auxin-related genes (69 up-regulated and 42 down-regulated; Supplementary Table S1) revealed that within the up-regulated genes, genes related to response, transport, metabolism, and auxin homeostasis were significantly altered in response to the Rp. Some of the up-regulated genes related to auxin transport were *PIN*1, *PIN*2, *PIN*3, *PIN*4, *PIN*7, *AUX*1, and *SAV*3; while IAA (i.e., *IAA*11, *IAA*30, *IAA*34) and SAUR-like genes were down-regulated (Supplementary Table S1). These findings confirm that auxins play a significant role in the light-mediated germination process, affecting its metabolism, signaling, and transport.

### 2.2. R-Light Alters Auxin Levels in Germinating Seeds

**Figure 2:**
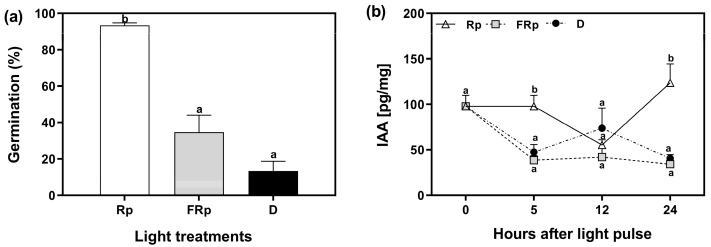
Auxin levels are induced by R-light in germinating seeds of *Arabidopsis thaliana*. (**a**) Col-0 seeds were sown for 2 h in darkness and then irradiated with an FR pulse (FRp). Seeds were then incubated at 5 °C in darkness for 72 h and finally irradiated with a red pulse (Rp) or an FRp. Germination was counted after 3 d at 25 °C. Each bar represents the mean ± SE (n = 6). (**b**) Quantification of IAA levels in seeds [pg/mg]. Each point represents the mean ± SE (n = 5). Different letters denote significant differences among means at the same time point (*p* < 0.05 by ANOVA followed by Tukey posttest). D: darkness; FRp: far-red pulse; Rp: red pulse.

To elucidate the role of auxins in seed germination, we quantified the levels of indole-3-acetic acid (IAA) in Col-0 seeds. The seeds were incubated for 72 h at 5 °C and subsequently exposed to saturating pulses of R or FR light, while keeping a dark control. The endogenous auxin levels were measured at specific time points: 0, 5, 12, and 24 h after the light pulses. The Rp induced 95% of germination, whereas the germination in response to the FRp was 34%, and 13% in darkness (Figure 2a). We used these seeds to quantify the levels of IAA. IAA levels were low when seeds received an FRp or were kept in darkness, and they remained relatively stable at 12 h and 24 h. The reduction in auxin levels by the FRp and darkness implies that auxin biosynthesis, conjugation, or degradation pathways could be related to phytochrome-mediated germination. Conversely, seeds irradiated with an Rp exhibited elevated auxin levels within the first 5 h, followed by a decline to low levels and levels comparable to those of FRp and dark conditions at 12 h. Notably, 24 h after the Rp, IAA levels were significantly higher than those found in FRp and darkness, nearly tripling in value. This result suggests that R-induced seed germination probably enhances auxin biosynthesis or reduces its conjugation/degradation (Figure 2b). Altogether, our data show that auxin levels in seeds that undergo R-induced germination display a biphasic response: (a) an early phase of high auxin levels between 0 and 5 h, likely associated with minimal degradation of the endogenous auxin levels synthetized during seed embryogenesis in the mother plant or during seed imbibition in cold; and (b) a late phase beginning at 12 h after the light pulse, which is possibly linked to de novo synthesis. In summary, our results suggest the existence of dynamic changes in auxin levels during R-light-induced seed germination.

### 2.3. Auxin Transport Is Required in Germinating Seeds

**Figure 3:**
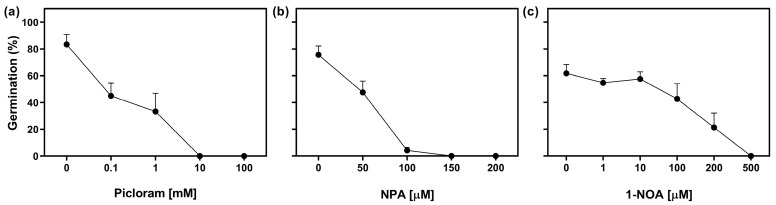
The promotion of germination by R-light requires the auxin transport. Col-0 seeds were sown in a medium supplemented with (**a**) Picloram, (**b**) N-1-naphthylphthalamic acid (NPA), and (**c**) 1-naphthoxyacetic acid (1-NOA) for 2 h in darkness and then irradiated with an FRp before the incubation at 5 °C in darkness for 3 d. Then the seeds were irradiated with an Rp and germination was counted after 3 d at 25 °C. Each point represents mean ± SE (n ≥ 6).

We evaluated the effect of exogenous auxin treatment on seed germination of Col-0 seeds by using increasing concentrations of Picloram (a synthetic auxin). Seeds were stratified for 3 days in darkness prior to the Rp. Doses ranging from 0 to 1 mM resulted in a 50% reduction in seed germination, while higher concentrations completely inhibited germination (Figure 3a). In addition, we explored the role of auxin transport by using polar auxin transport inhibitors, namely N-1-naphthylphthalamic acid (NPA), an auxin efflux inhibitor, and 1-naphthoxyacetic acid (1-NOA), an auxin influx inhibitor [25]. The imbibition of seeds in NPA showed a significant impact on germination. At a concentration of 50 μM, germination was reduced by 35%, and concentrations equal to or above 100 μM completely inhibited germination (Figure 3b). On the other hand, the addition of doses between 0 μM and 10 μM of 1-NOA had no effect on seed germination. However, at higher concentrations, germination was progressively reduced, and completely inhibited at 500 μM of 1-NOA (Figure 3c). These results strongly suggest that the regulation of auxin distribution through auxin transporters could play a significant role in R-light seed germination and that the increased auxin negatively regulates the germination of Arabidopsis seeds.

### 2.4. PIN3, PIN7, and AUX1 Are Involved in Seed Germination

**Figure 4:**
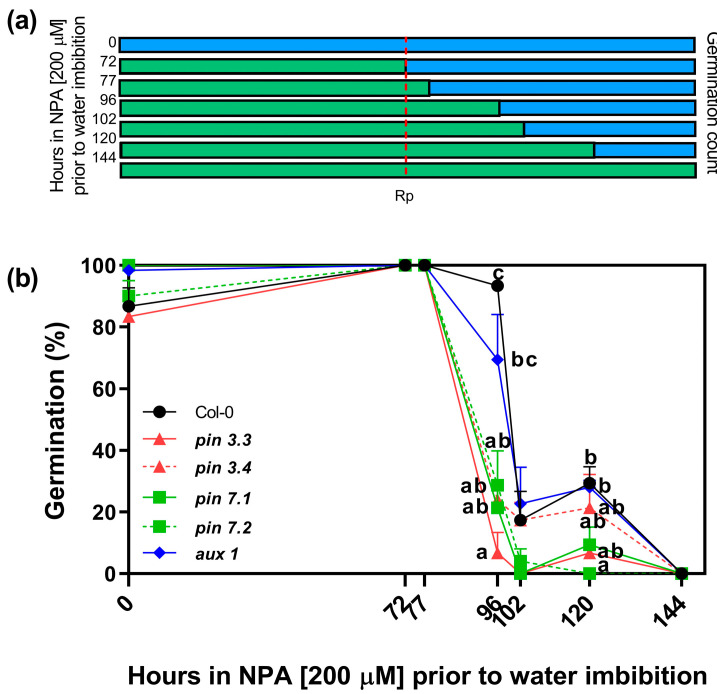
Effect of prolonged incubation with NPA on seed germination. (**a**) Scheme of the experimental protocol. Seeds were incubated for 2 h in darkness and then irradiated with an FRp. Seeds were then incubated for 3 days at 5 °C and irradiated with an Rp. Germination was counted after 3 days at 25 °C. The blue bars represent the incubation period in water and the green bars represent the incubation period in NPA 200 μM. (**b**) Germination percentage of Col-0 and auxin carriers mutant seeds in response to an Rp. The incubation medium was supplemented with NPA 200 μM for variable periods after seed imbibition (0 h, 72 h, 77 h, 96 h, 102 h, 120 h, and 144 h), keeping a control in water (0 h) and a control in NPA 200 μM (144 h). Each point represents the mean ± SE (n ≥ 6). Different letters denote significant differences among means at the same time point (*p* < 0.05 by ANOVA followed by Tukey posttest). Rp: red pulse.

Our previous results suggest that R-treated seeds have increased endogenous levels of auxins (Figure 2) and that increased levels of auxins (by the addition of picloram), and inhibition of auxin transport (by the addition of the auxin transport inhibitors NPA and 1-NOA), can inhibit seed germination (Figure 3). Thus, we evaluated the effects of the NPA applied in the incubation medium before and after irradiating Col-0 seeds with an Rp. The rationale of this experimental design was to evaluate the time-course in which the auxin transport is relevant to induce seed germination. Seeds were imbibed in NPA 200 μM and stratified for 3 days prior to the Rp. Seeds were then transferred to a medium containing distilled water at different time points (72, 77, 96, 102, 120 h). We also added positive and negative controls by imbibing seeds solely in water (0 h) or with NPA during all the experiment (144 h, Figure 4a). Seeds kept in darkness or irradiated with an FRp did not germinate (Supplementary Figure S1). The germination of Col-0 seeds irradiated with an Rp was ~80% in water and 0% in NPA 200 μM (0 h and 144 h, respectively; Figure 4b). The germination levels of seeds imbibed with NPA 200 μM up to 24 h after the Rp (96 h) was not altered, but longer periods of incubation in NPA 200 μM (102 h and 120 h) reduced germination to ~20% in Col-0 seeds. These results strongly suggest that auxin transport plays a crucial role after the 24 h induction of seed germination by the Rp, when the auxin levels in the seeds are increased (Figure 2b). 

Furthermore, we investigated the involvement of the specific auxin efflux (PIN3 and PIN7) and influx (AUX1) carriers under the same experimental conditions. To achieve this, we analyzed the germination responses of two null mutant alleles of PIN3 (*pin*3.3, *pin*3.4), two null mutant alleles of PIN7 (*pin*7.1, *pin*7.2), and one null mutant allele of AUX1 (*aux*1.1) seeds (Figure 4b). When NPA 200 μM was applied in the incubation medium, we observed that all mutant seeds exhibited greater sensitivity to NPA compared to Col-0. Notably, *pin*3.3 mutant seeds did not germinate when NPA 200 μM was maintained in the incubation medium for 24 h after the R-light pulse (96 h; as shown in Figure 4b). Additionally, *pin*3.4, *pin*7.1, and *pin*7.2 mutant seeds exhibited a significant reduction in germination compared to Col-0, suggesting that the absence of PIN3 and PIN7 has a strong impact on germination. In the case of *aux*1.1 seeds, a longer imbibition period was required to suppress germination (120 h). These data demonstrate that auxin transport plays a pivotal role during R-light-induced seed germination, and suggest that PIN3, PIN7, and AUX1 are relevant auxin transporters required for the promotion of seed germination by light. 

### 2.5. ABA Inhibits the Activity of PIN3, PIN7, and AUX1

**Figure 5:**
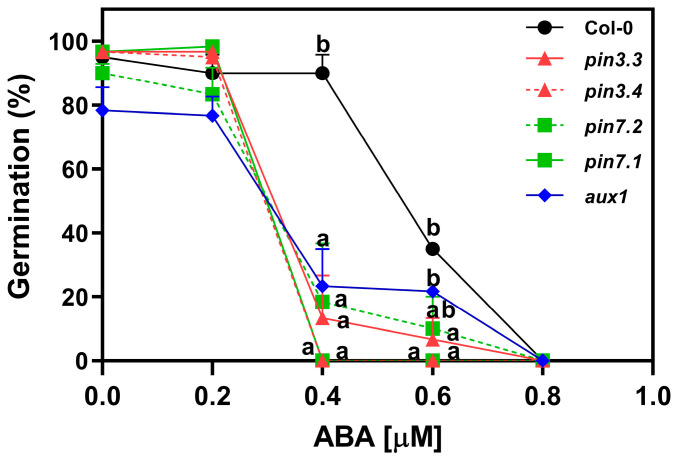
ABA inhibits the activity of PIN3, PIN7, and AUX1 in *Arabidopsis thaliana* seeds. Germination of Col-0, *pin*3.3, *pin*3.4, *pin*7.1, *pin*7.2, and *aux*1.1 seeds incubated for 72 h at 5 °C, before the Rp. Seeds were imbibed with increasing doses of ABA. Each point represents the mean ± SE (n ≥ 6). Different letters denote significant differences among means at the same ABA concentration (*p* < 0.05 by ANOVA followed by Tukey posttest).

To investigate the influence of auxin transport on abscisic acid (ABA) sensitivity, we analyzed the germination of R-treated seeds imbibed in increasing concentrations of ABA supplemented with Fluridone (F) 100 μM, an inhibitor of ABA synthesis. The germination of Col-0 seeds was ~90% at concentrations ranging from ABA 0 μM to 0.4 μM. The addition of 0.6 μM ABA reduced the germination by 65%, and 0.8 μM ABA completely inhibited the germination of Col-0 seeds (Figure 5). *pin*3.3, *pin*3.4, *pin*7.1, *pin*7.2, and *aux*1.1 seeds, on the other hand, exhibited a drastic reduction in germination at 0.4 μM ABA. This heightened sensitivity to ABA indicates that the absence of PIN3, PIN7, and AUX1 significantly influences the seeds’ response to the inhibitory effects of ABA on germination. This suggests that both functional auxin efflux (at least through PIN3 and PIN7) as well as auxin influx (at least through AUX1) transport are necessary for exerting the ABA’s inhibitory effects on seed germination. 

### 2.6. Red-Light Perception and ABA Alter the Expression of PIN3, PIN7, and AUX1

**Figure 6:**
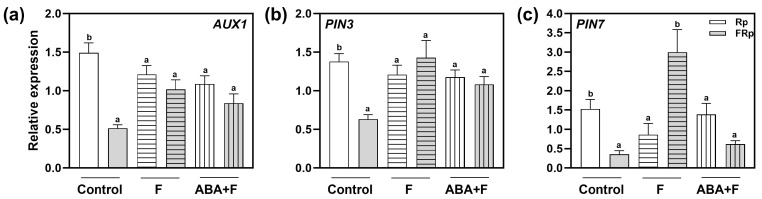
Red-light regulates the expression of *AUX*1, *PIN*3, and *PIN*7. Gene expression levels of (**a**) *AUX*1, (**b**) *PIN*3, and (**c**) *PIN*7 were normalized to *PP*2*A*. Col-0 seeds were imbibed in water (control), fluridone (F) 100 mM, or ABA 1 mM supplemented with F 100 mM (ABA+F) and irradiated with an Rp or an FRp. Seed samples were harvested 12 h after the pulses. Each bar represents mean ± SE (n = 3). Different letters denote significant differences among means at the same imbibition treatment (*p* < 0.05 by Student’s *t*-test). FRp: far-red pulse; Rp: red pulse.

The interactions between auxin and ABA regulate many developmental processes, including seed germination [26]. Since we showed that the auxin transporters PIN3, PIN7, and AUX1 are key components connecting the ABA and auxin signaling cascades, we evaluated whether their gene expression might be altered in response to ABA. To this end, we analyzed the gene expression of *PIN*3, *PIN*7, and *AUX*1 when Col-0 seeds were imbibed in water (control), Fluridone (F), and F supplemented with ABA (ABA+F) (Figure 6). Rp-treated Col-0 seeds significantly increased the expression of *AUX*1, *PIN*7, and *PIN*3 between 1- and 1.5-folds when compared to the FRp (Figure 6), confirming that the expression of these genes is modulated by R-light. Interestingly, the absence of ABA in the seeds (by the addition of F) abolished the changes in the gene expression of *AUX1* and *PIN*3 (Figure 6a,b) since the FRp increased their expression at similar levels to those exposed to the Rp. These results suggest a direct ABA-inhibitory effect on the light-mediated gene expression of these auxin transporters. Furthermore, *PIN*7 expression is significantly promoted by an FRp and inhibited by an Rp (Figure 6c), suggesting a complex regulatory mechanism in this specific auxin transporter in response to light and ABA. Finally, the expression of auxin transporter genes promoted by an Rp disappeared during the ABA + F treatment (Figure 6a–c), indicating that the ABA sensitivity also abolishes *AUX1*, *PIN3*, and *PIN7* light-induced gene expression. 

## 3. Discussion

Our results highlight the importance of auxin transport in Arabidopsis seed germination promoted by light. We demonstrated that R-light, which induces seed germination, maintains high levels of active auxin immediately after light stimulation, between 0 and 5 h, and then increases the auxin levels at 24 h (Figure 2). Between 5 and 12 h after the Rp, there is a significant reduction in auxin levels in the seed tissues showing a biphasic response. These results suggest that the endogenous auxins from the early phase are the ones formed during seed maturation in the mother plant, and the auxin levels reached in the late phase are the result of “de novo” synthesis occurring after seed imbibition. Indole-3-acetic acid (IAA) in dry seeds is mainly stored as conjugates, which are hydrolyzed during early imbibition to yield free IAA [27]. The hydrolysis of the IAA conjugates is a rapid process that allows immediate access of IAA and removes the need for long-distance transport. This process is followed by tryptophan-dependent auxin biosynthesis in developing tissues in the root and shoot apex [28]. The increase in IAA in imbibed seeds 24 h after the Rp suggests that high levels of auxin are a prerequisite important for seed germination and to further promote the elongation of the embryonic axis, leading to the radicle protrusion. Under our experimental conditions, when seeds are imbibed in water, germination is completed within 36 h after the Rp, and this result also agrees with that obtained from seeds exposed to white light [29]. 

Furthermore, we showed that auxin transport is relevant for R-light-induced germination (Figure 3, Figure 4 and Figure 5) and likely plays a role in maintaining high levels of auxin in seed tissues, which are essential for the initiation of germination. Our results show that the germination is repressed when Arabidopsis seeds are imbibed in a medium with 1-NOA or NPA, which represses the activity of auxin influx and efflux transporters, and the effects are similar to the addition of high doses of synthetic auxins (Figure 3). Additionally, we showed that R-light-induced germination is inhibited in *pin*3, *pin*7, and *aux*1 null mutant seeds, demonstrating that these auxin efflux and influx protein carriers, respectively, are necessary to promote germination (Figure 4). Interestingly, the auxin influx carrier AUX1 was recently reported to have a positive role in seed germination regulated by histone H3K9K18 deacetylation. AUX1 has been involved in the radicle growth of germinating seeds by facilitating the transport and accumulation of auxins in the tip of the radicle [30].

Light-induced germination results from the integration of the signaling networks of different hormones (Figure 1). The repressive action of ABA on seed germination is counteracted by the presence of PIN3, PIN7, and AUX1 transporters (Figure 5). The altered sensitivity to ABA in the mutant seeds underscores the relevance of PIN3, PIN7, and AUX1 in maintaining a proper germination in response to ABA, highlighting a possible interplay between auxin transport and ABA signaling on seed germination (Figure 5). Previous works have documented the crosstalk between auxins and ABA in imbibed Arabidopsis seeds. For example, *arf*10 mutant seeds are hypersensitive to ABA, and MIR160 overexpression counteracts the inhibitory effects of ABA, suggesting a negative regulation of ARF10 by miR160 when seeds are imbibed in ABA. Contrarily, there are other reports showing positive effects between auxins and ABA on dormancy and post-germination in Arabidopsis seeds [12]. Some reports documented a positive correlation between auxin content/signaling in seed dormancy. Seeds that overexpress auxin content (iaaM-OX) seeds exhibit strong dormancy, while *yuc*1*/yuc*6, *tir*1*/afb*3, and *tir*1*/afb*2 mutant seeds show reduced seed dormancy. Auxins can induce dormancy by enhancing ABA sensitivity through the recruitment of *ARF*10/16 to maintain *ABI*3 expression in Arabidopsis seeds [18]. Moreover, under unfavorable environmental conditions, ABA inhibits the radicle and hypocotyl growth in cooperation with auxins [22]. Auxin flows in the epidermis and cortex of the axis elongation zone are critical to repress elongation in response to ABA, and *aux*1 and *pin*2 mutants are insensitive to ABA-dependent repression of embryonic axis elongation [22]. ABA represses embryonic axis elongation by enhancing auxin signaling, which involves the repression of AXR2/IAA7 in the cell elongation zone. Taken together, the effects of auxin and ABA crosstalk are dependent on the seed status: auxins promote seed dormancy when ABA levels in the seeds are high and repress radicle and hypocotyl elongation under stress conditions mediated by ABA during post-germination events. In opposition, the results from our work suggest that auxin is relevant in light-mediated seed germination, and its action involves mechanisms associated with auxin transporters which are inhibited by ABA. 

We have shown that *PIN3*, *PIN7*, and *AUX1* expression is promoted by R-light, and ABA affects their expression (Figure 5 and Figure 6). Indeed, the imbibition of seeds with ABA supplemented with fluridone (ABA+F) abolished the Rp-dependent expression of *PIN*3, *PIN*7, and *AUX*1 *(*Figure 6). Interestingly, *PIN*7 expression was three folds higher in FRp-treated seeds compared to the Rp-treated ones when seeds were imbibed with fluridone alone, suggesting that the auxin transporter’s expression involves complex and opposite regulatory mechanisms mediated by light. These results are in accordance with previous transcriptomes in germinating seeds that revealed up- and down-regulation of *AUX*1, *PIN*2, and *PIN*7 genes in response to GA and ABA, respectively [31,32,33]. In germinating seeds, the ABA levels are low, and the auxin levels increase; thus, auxin is redistributed by the auxin transporters into seed tissues to promote the growth of the radicle. The increase in *PIN*3, *PIN*7, and *AUX*1 gene expression in R-induced germinating seeds might lead to the relocation of their proteins to the epidermal cells within the embryonic axis elongation zone. This process would enhance auxin transport, resulting in elevated auxin levels directed towards the tip of the embryo’s radicle. Consequently, this enhanced auxin distribution would promote cell elongation, ultimately facilitating endosperm rupture for seed germination. A similar mode of action has been shown for PIN2 and AUX1 through the repression of *AXR*2*/IAA*7 and *AXR*3*/IAA*17 expression in ABA-treated Arabidopsis seeds [22]. Also, a comparable mechanism involving PIN3 has been proposed for Arabidopsis seedlings exposed to shade light, where the low R:FR ratio induces changes in auxin distribution in hypocotyls by relocating PIN3 within the tissues to enhance the stem elongation growth under dense canopies [34]. Further studies could unravel the precise mechanisms underlying the expression and activity patterns of auxin transporters in seeds exposed to different light conditions. Interestingly, ABI4 reduces the concentration of auxin in the primary root tip by suppressing the expression of *PIN*1 [35]. In this sense, we cannot discard the reality that, under our experimental conditions, ABI4 could also be altering *PIN*3 and *PIN*7 expression through its impact on ABI4-mediated auxin pathways.

## 4. Materials and Methods

### 4.1. Plant Material and Growth Conditions

*Arabidopsis thaliana* plants were grown as previously described in [24]. Plants were grown under long day conditions (16 h light/8 h dark, PAR = 100 µmol m^−2^ s^−1^) with an average temperature of 21 ± 2 °C. Wild-type and mutant plants were grown together and their mature seeds were harvested at the same time to avoid differences in post-maturation that can affect seed germination. Seeds of each genotype were harvested as a single bulk consisting of at least 5 plants. Seeds were stored in open tubes inside a closed box and maintained in darkness using silica gel at 4 °C until the experiments were performed. *Arabidopsis thaliana* wild-type seeds were Columbia-0 (Col-0). In this study, we used insertional knock-out lines: *pin*3.3 [36], *pin*3.4 (Salk_038609), *pin*7.1 (Salk_044687C), *pin*7.2 (CS9366), and *aux*1 (CS859699).

### 4.2. Germination Conditions and Light Treatments

Samples of 30 seeds per genotype were sown in clear plastic boxes (40 × 33 × 15 mm), each containing 10 mL of 0.8% (*w*/*v*) agar in demineralized water. To establish a minimum and equal photo-equilibrium, seeds were imbibed for 2 h in darkness and then irradiated for 20 min with a saturated far-red pulse (FRp; calculated Pfr/P = 0.03, 42 µmol·m^−2^·s^−1^) to minimize the quantities of Pfr that formed during their development in the mother plant. Seeds were then stratified at 5 °C in darkness for 3 days, prior to the 20 min with a saturated Rp (calculated Pfr/P = 0.87, 35 µmol·m^−2^·s^−1^) or FRp. After light treatments, the boxes containing the seeds were wrapped again with black plastic bags and incubated at 25 °C for 3 days before germination was determined. The criterion for germination was the emergence of the radicle.

For the germination assays where hormones were used, 6 biological replicates of 30 seeds were sown in clear plastic boxes (40 × 33 × 15 mm), each containing filter papers imbibed with 750 μL of 1-NOA (1 μM, 10 μM, 100 μM, 200 μM, and 500 μM), NPA (50 μM, 100 μM, 150 μM, and 200 μM), Picloram (0.1 mM, 1 mM, 10 mM, and 100 mM), and ABA (0.2 μM, 0.4 μM, 0.6 μM, and 0.8 μM) (Sigma-Aldrich, Steinheim, Germany).

### 4.3. Window of Sensitivity to NPA

Six biological replicates of 30 seeds each were sown onto filter papers in plastic boxes (40 × 33 × 15 mm). The filter papers were soaked with 750 μL of hormonal solution or water, depending on the experiment phase. Seeds soaked in a 200 μM NPA solution were imbibed for 2 h in darkness and then irradiated for 20 min with a saturated FRp (calculated Pfr/P = 0.03, 42 µmol·m^−2^·s^−1^) to minimize the quantities of Pfr that formed during their development in the mother plant. Seeds were then stratified at 5 °C in darkness for 3 days, prior to the 20 min with a saturated Rp (calculated Pfr/P = 0.87, 35 µmol·m^−2^·s^−1^) or FRp. After the Rp, the seeds on filter papers soaked with NPA were transferred to new filter papers soaked in distilled water at different time-points (0 h, 5 h, 24 h, 30 h, and 48 h). We also kept water and NPA controls. Changes in the solutions were performed under dim green safe light. 

### 4.4. Quantification of IAA Levels

Six biological replicates of seeds were used for each light treatment. Samples for IAA quantification (5–10 mg fresh weight) were collected 0, 5, 12, and 24 h after irradiation with the R and FR pulse, along with the corresponding dark controls. Each sample was homogenized in sodium phosphate buffer containing 1 ng ^13^C_6_-IAA internal standard, purified, and quantified according to [37]. IAA levels were measured using gas chromatography-selected reaction-monitoring mass spectrometry.

### 4.5. cDNA Library Preparation and High-Throughput Sequencing, Processing of RNA Sequencing Reads, and Differential Gene Expression Analysis

This has been originally described in [24]. The RNA-seq data from [24] is available at the Gene Expression Omnibus (GEO) with this accession: GSE134019.

### 4.6. Gene Expression Analysis by Quantitative RT–PCR

Seed samples (~5 mg seed dry weight) were sown in clear plastic boxes, each containing 10 mL of 0.8% (*w*/*v*) agar in demineralized water and incubated for three days in darkness at 5 °C. Seeds were then irradiated with an Rp or FRp and samples were collected at 12 h upon light irradiation. After sampling, seeds were immediately frozen in liquid nitrogen and stored at − 80 °C. RNA was extracted using the Spectrum Plant Total RNA Kit (Sigma-Aldrich, Steinheim, Germany) according to the manufacturer’s protocol. cDNA was synthesized using M-MLV Reverse Transcriptase (Sigma-Aldrich, Steinheim, Germany) and oligo-dT primers. The synthesized cDNAs were amplified with Master Mix qPCR 2X with SYBR (Inbio Highway, Tandil, Argentina) using the QuantStudio^TM^3 (Applied Biosystems, Foster City, CA, USA). *PP*2*A* gene was used as a normalization control. The primers used are described in Supplementary Table S2.

### 4.7. Experimental Design and Statistical Analysis

Physiological and hormonal experiments were performed using at least three different populations of seeds harvested for at least 5 plants. To test for significant differences in the response of the seeds, we conducted two-way analyses of variance (ANOVAs) for each WT and mutant group, using the angular transformation of the percentage of germination and the InfoStat Software version 2017 (Grupo InfoStat, FCA, Universidad Nacional de Córdoba, Argentina). Tukey post-test was used to test differences between genotypes when significant treatment-by-genotype interactions were observed. To test for significant differences in the gene expression, we conducted Student’s *t*-test.

## Data Availability

The authors confirm that the data supporting the findings of this study are available within the article and its Supplementary Materials.

## Supplementary Materials

The following supporting information can be downloaded at: https://www.mdpi.com/article/10.3390/plants13030408/s1. Supplementary Figure S1: Effect of prolonged incubation with NPA on the germination of FR-treated seeds and seeds kept in darkness. Supplementary Table S1: Differential gene expression analyses. Supplementary Table S2: Primers used in the study.
