# Supplementary figures and images for: Unveiling Molecular Signatures in Light-Induced Seed Germination: Insights from PIN3, PIN7, and AUX1 in Arabidopsis thaliana
Source: Plants (Basel). 2024 Jan 30;13(3):408. doi: 10.3390/plants13030408 (PMC10856848; doi:10.3390/plants13030408)

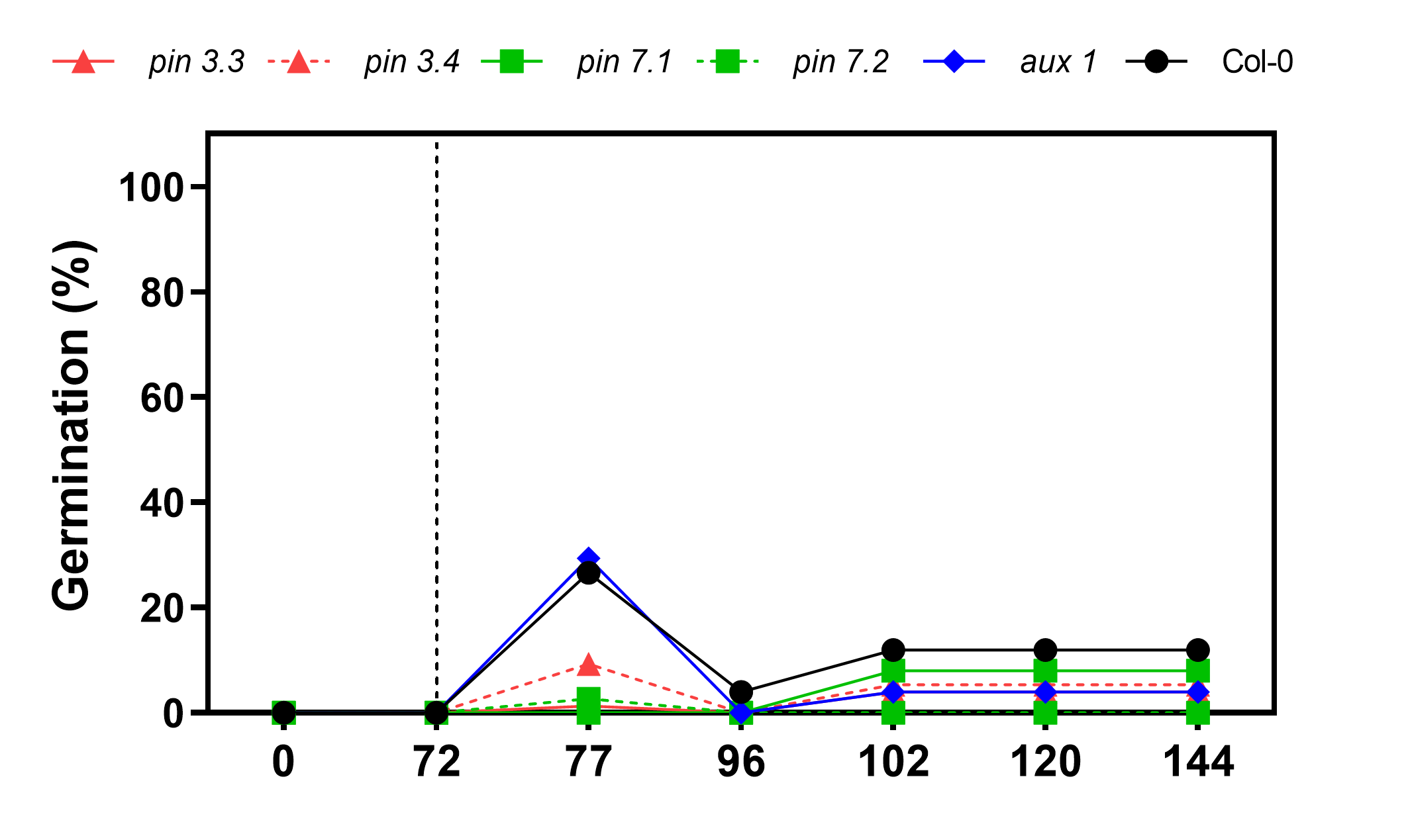

Supplement: Supplementary file 1 [file plants-13-00408-s001.zip › Supplementary_Figure_S1.tif]
